# Comparative Genomic Analysis Identifies Divergent Genomic Features of Pathogenic *Enterococcus cecorum* Including a Type IC CRISPR-Cas System, a Capsule Locus, an *epa*-Like Locus, and Putative Host Tissue Binding Proteins

**DOI:** 10.1371/journal.pone.0121294

**Published:** 2015-04-10

**Authors:** Luke B. Borst, M. Mitsu Suyemoto, Elizabeth H. Scholl, Fredrick J. Fuller, H. John Barnes

**Affiliations:** 1 Department of Population Health and Pathobiology, College of Veterinary Medicine, North Carolina State University, Raleigh, North Carolina, United States of America; 2 Bioinformatics Consulting and Service Core, Bioinformatics Research Center, College of Agriculture and Life Sciences, College of Sciences, North Carolina State University, Raleigh, North Carolina, United States of America; University of Kansas, UNITED STATES

## Abstract

*Enterococcus cecorum* (EC) is the dominant enteric commensal of adult chickens and contributes to the gut consortia of many avian and mammalian species. While EC infection is an uncommon zoonosis, like other enterococcal species it can cause life-threating nosocomial infection in people. In contrast to other enterococci which are considered opportunistic pathogens, emerging pathogenic strains of EC cause outbreaks of musculoskeletal disease in broiler chickens. Typical morbidity and mortality is comparable to other important infectious diseases of poultry. In molecular epidemiologic studies, pathogenic EC strains were found to be genetically clonal. These findings suggested acquisition of specific virulence determinants by pathogenic EC. To identify divergent genomic features and acquired virulence determinants in pathogenic EC; comparative genomic analysis was performed on genomes of 3 pathogenic and 3 commensal strains of EC. Pathogenic isolates had smaller genomes with a higher GC content, and they demonstrated large regions of synteny compared to commensal isolates. A molecular phylogenetic analysis demonstrated sequence divergence in pathogenic EC genomes. At a threshold of 98% identity, 414 predicted proteins were identified that were highly conserved in pathogenic EC but not in commensal EC. Among these, divergent CRISPR-*cas* defense loci were observed. In commensal EC, the type IIA arrangement typical for enterococci was present; however, pathogenic EC had a type IC locus, which is novel in enterococci but commonly observed in streptococci. Potential mediators of virulence identified in this analysis included a polysaccharide capsular locus similar to that recently described for *E*. *faecium*, an *epa*-like locus, and cell wall associated proteins which may bind host extracellular matrix. This analysis identified specific genomic regions, coding sequences, and predicted proteins which may be related to the divergent evolution and increased virulence of emerging pathogenic strains of EC.

## Introduction

Until recently, the only recognized role of *Enterococcus cecorum* (EC) in health and disease was as an enteric commensal of adult chickens and a rare cause of systemic infection in humans.[1–7] EC has been recovered from the intestinal tracts of both birds and mammals including chickens, ducks, horses, calves, pigs, cats, and dogs.[3, 4, 8] In chickens, EC becomes the dominant commensal of adult birds by 12 weeks of age.[3, 8] However, since 2002, emerging pathogenic strains of EC have been responsible for outbreaks of musculoskeletal disease in broiler and broiler-breeder chickens worldwide.[9–19] EC-associated disease outbreaks were first documented in broiler flocks in Europe and outbreaks with similar clinical presentation, gross findings, and epidemiology have since been reported in North America.[10, 12, 15, 16, 20, 21] In the US, outbreaks of EC-associated disease have been reported in 10 states including all of the 5 top broiler chicken producing states: Alabama, Arkansas, Georgia, Mississippi, and North Carolina.[9, 14, 17–19, 22, 23] Recently, pathogenic EC has demonstrated an expanded host range to include Pekin ducks.[24]

Pathogenic EC causes the disease, Enterococcal Spondylitis (ES).[17, 25] In ES, chronic infection of the free thoracic vertebra (FTV) by pathogenic EC leads to compression of the overlying spinal cord resulting in paralysis.[9, 14, 17, 25] Clinical signs of paralysis appear in affected birds as they near market weight at 4–8 weeks and outbreak-associated mortality due to culling or dehydration/starvation of paralyzed birds can approach 15%.[9, 14, 17–19, 22, 23]

Pathogenic EC have been shown to be genetically clonal. In a prior work we reported a molecular epidemiologic investigation of 22 epidemiologically distinct outbreaks of ES in the southeast US.[9] In this study, commensal EC recovered from the intestines of affected and unaffected control birds were compared with pathogenic EC recovered from spinal lesions. While commensal isolates were found to exhibit genetic diversity; with the exception of a single cluster of strains from one outbreak, spinal isolates tightly clustered into two major clusters separate from the commensal strains. In addition to being genetically distinct from commensal EC, pathogenic EC genotypes were found to be highly similar to each other. At a cut-off of >72% similarity two clusters of pathogenic EC were observed. However, at >92% similarity only 3 clusters form within the pathogenic EC group.

Phenotypically, pathogenic EC isolates were found to be deficient in mannitol metabolism while commensal EC isolates are able to metabolize mannitol.[9] However, spinal isolates were found to be significantly more drug resistant than commensal strains with 90–100% of pathogenic strains resistant to macrolides, tetracyclines, and sulfonamides.[9] Finally, isolates with pathogenic genotypes were found to be significantly more virulent than commensal EC in a chicken embryo lethality model.[26]

The clonality and the increased virulence of strains isolated from spinal lesions suggest that pathogenic EC strains have acquired specific virulence determinants. Therefore the goals of this study were 1) to sequence and compare the general features of commensal and pathogenic EC genomes and 2) to identify potential virulence genes that are present in pathogenic EC but not in commensal EC. For this analysis 3 pathogenic EC strains representing the 3 clusters observed at >92% similarity in our prior study and 3 commensal EC isolates were selected for *de novo* genome assembly. Genomes were compared to identify coding sequences (CDS) and predicted proteins that are not present or highly conserved in commensal EC but present and highly conserved in pathogenic EC. Our analyses revealed several features unique to pathogenic EC including regions of gene acquisition and loss, divergent CRISPR-*cas* defense loci and putative virulence determinants which may play a critical role in the recently acquired virulence of pathogenic EC.

## Materials and Methods

### Bacterial Strains

In a prior study, genotypes of pathogenic EC were determined using pulsed-field gel electrophoresis (PFGE) and compared using the Unweighted Pair Group Method with Arithmetic Mean (UPGMA).[9] With the exception of isolates from a single outbreak, genotypes of pathogenic EC fell into 2 major clusters at >72% similarity and 3 clusters at >92% similarity. Representative strains SA1, SA2, and SA3 were selected to represent these 3 clusters at >92% similarity. SA1 and SA2 are found within one large cluster at 72% similarity but in 2 separate clusters at 92% similarity. SA3 represents the second large cluster of pathogenic EC which is separate from SA1 and SA2 at both 72% and 92% similarity. SA1, SA2, and SA3 were isolated from spinal lesions of affected birds during epidemiologically distinct outbreak investigations from 3 different NC integrated poultry companies which occurred in 2010 (SA1 and SA2) and 2011 (SA3). Commensal isolates CE1, CE2, and CE3 were isolated from ceca of unaffected birds during this same period. CE1, CE2, and CE3 have diverse genotypes but all cluster with commensal strains. When evaluated for virulence in a chicken embryo lethality assay, SA1, SA2, and SA3 were significantly more virulent than CE1, CE2, and CE3.[26]

Bacteria were grown on trypticase soy agar with 5% sheep blood or in Todd-Hewitt Broth with 1.0% yeast extract (THBY) at 37°C with 5% CO_2_. Whole genomic DNA was obtained using a commercial kit according to the manufacturer’s instructions (MasterPure Gram Positive DNA Purification Kit, Epicentre). PFGE of undigested genomic DNA was utilized to confirm the absence of large extra-chromosomal genetic elements in all strains prior to sequencing.

### Genome Assembly and Comparison

The Illumina GAIIx was used to sequence SA1 and Illumina HiSeq was used to sequence the remaining 5 strains. The first 15 bases of the reads for all data sets were trimmed using the FASTX-Toolkit (http://hannonlab.cshl.edu/fastx_toolkit/index.html) to remove sequence noise. Due to the large number of reads for SA2, SA3, CE1, CE2, and CE3, a sample of 40 million paired reads was assembled for SA2 and a sample of 80 million paired reads was assembled for the remaining strains. Sequence assembly for all strains was accomplished using the CLC Bio Genomics Workbench (Cambridge, Massachusetts) with settings for paired-end reads and scaffolding. To create a single chromosome sequence for each of the samples, scaffolds were ordered and oriented based on alignments to a recently published genomic sequence (ASWI01) of the ATCC type strain *Enterococcus cecorum* DSM 20682 (ATCC 43198). This strain was recovered from the ceca of a healthy chicken and represents the first description of EC, then named *Streptococcus cecorum*.[27] Sequence data were deposited into Genbank under BioProject PRJNA268097 (see Table 1). Chromosome length and GC content were calculated and pathogenic and commensal group values were compared using a two-tailed Student’s T test (Excel, Microsoft).

**Table 1:** General Genomic Features of Pathogenic and Commensal EC.

Strain	GenBank Accession*	Reads	Scaffolds	Largest Scaffold	Scaffold N50	Length (bp)	GC Content (%)	Total CDS
SA1	CP010060	39,928,574	48	172,331	112,924	2,261,297	36.5	2238
SA2	CP010061	40,000,000	47	172,332	128,936	2,281,990	36.5	2260
SA3	CP010064	89,466,410	752	261,845	128,731	2,320,621	36.0	2316
CE1	CP010059	102,538,988	679	194,734	47,277	2,438,959	35.7	2328
CE2	CP010062	98,387,538	859	231,280	50,626	2,437,337	35.7	2311
CE3	CP010063	83,921,612	678	153,548	47,016	2,372,217	35.8	2255

* All BioSamples are registered under BioProject PRJNA268097

Comparison of genomic rearrangement patterns among strains was performed using the following programs: MUMmer3.0, Artemis Comparison Tool (ACT) and Blast Ring Image Generator (BRIG) with BLASTn set at a minimum identity of 95%, with low complexity filtering turned off and an expected threshold = 1e^-5^ unless otherwise indicated.[28–30] For genomic comparisons SA3 and CE1 were established as reference genomes as they were the largest genomes of the pathogenic and commensal type, respectively. Using BLASTn in BRIG, all EC genomes were compared against SA3 and CE1 (including a comparison of SA3 and CE1 to themselves, as controls). For pathogenic strains, regions with <50% homology to commensal strains were selected for further analysis. The sequence for each of these regions was extracted from the reference sequence SA3 and compared to the NCBI Reference Sequence Database to identify genes and compared to the CE genome assemblies to verify that the genes did not exist in a different configuration in the commensal strains. ACT plots of these regions were constructed to detect genetic rearrangements.

### Comparison of Predicted Protein Sequence Identity

Assembled genomes were uploaded as single contigs in FASTA format to Rapid Annotation using Subsystems Technology (RAST) servers for annotation and compared using the SEED viewer.[31, 32] The Compare Sequence feature of the SEED viewer was used to compare sequence identity of the annotated predicted proteins of all strains.

To identify predicted proteins which were conserved in pathogenic strains, a stringent threshold of 98% protein sequence identity was used to filter predicted protein sequences. This stringent threshold was selected because of the clonal nature of pathogenic EC. Predicted proteins with >98% sequence identity in SA1, SA2, and SA3 and <98% identity in CE1, CE2, and CE3 were categorized as highly conserved in pathogenic EC and not highly conserved in commensal EC. To identify predicted proteins conserved in the more genetically diverse commensal EC, a less stringent threshold of 90% predicted protein sequence identity was used. Predicted proteins with >90% sequence identity in CE1, CE2, and CE3 and <90% identity in SA1, SA2, and SA3 were categorized as conserved in commensal EC.

Chromosomal regions containing conserved predicted proteins identified by these analyses were further investigated using the Compare Regions function of the SEED viewer. This function allowed for visualization of the annotated chromosomal arrangement surrounding proteins of interest in 4 or more similar organisms, including *E*. *faecalis* V583.

### Molecular Phylogenetic Analysis

A phylogenetic analysis of all 6 EC strains and *E*. *faecalis* V583 (as an outgroup) was performed using alignments of concatenated sequence of 5 genes that have been previously reported to be useful for speciation and classification of enterococci: 16S rDNA (*rrs*), D-ala:D-ala ligase (*ddl*), the manganese-dependent superoxide dismutase (*sodA*), chaperonin 60 (*cpn60*), and the alpha subunit of ATP synthase (*atpA*).[33–37] Sequence of these 5 genes was identified in all 7 enterococcal strains and individual alignments were performed to confirm genes were orthologs while assessing for any poorly conserved regions. The gene sequences were concatenated for each strain, aligned and a phylogenetic tree was created using the maximum likelihood method based on the Tamura-Nei model in MEGA6 for the 6 EC strains with *E*. *faecalis* V583 as an outgroup.[38, 39]

## Results and Discussion

### Divergent EC Genome Features

General genomic features for pathogenic and commensal EC are provided in Table 1. EC genomes ranged in size from 2,261,297 to 2,438,959bp and had GC contents ranging from 35.7–36.5%. The genome of pathogenic EC strains was significantly smaller (p = 0.01) compared to commensal EC strains. Pathogenic EC genomes averaged 128,202bp less than the average size of commensal strain genomes. The GC content of the pathogenic strains (36.0–36.5%) was significantly (p = 0.02) higher compared to commensal strains (35.7–35.8%). While the SA3 genome does have an approximately 400kb region of decreased GC content and skew; increased GC content does not appear to be regionally distributed within the genome (see S1 Fig). In general, genome size and GC content are similar to those observed in other streptococcal and enterococcal genomes.[8, 40–44] Number of CDS per genome was similar averaging 2,271 for pathogenic strains and 2,298 for commensal strains. Pathogenic strains contained genomic islands with Tn916-like genes and tetracycline resistance genes; however, the genomic location of this island varied among pathogenic strains.

**Fig 1:**
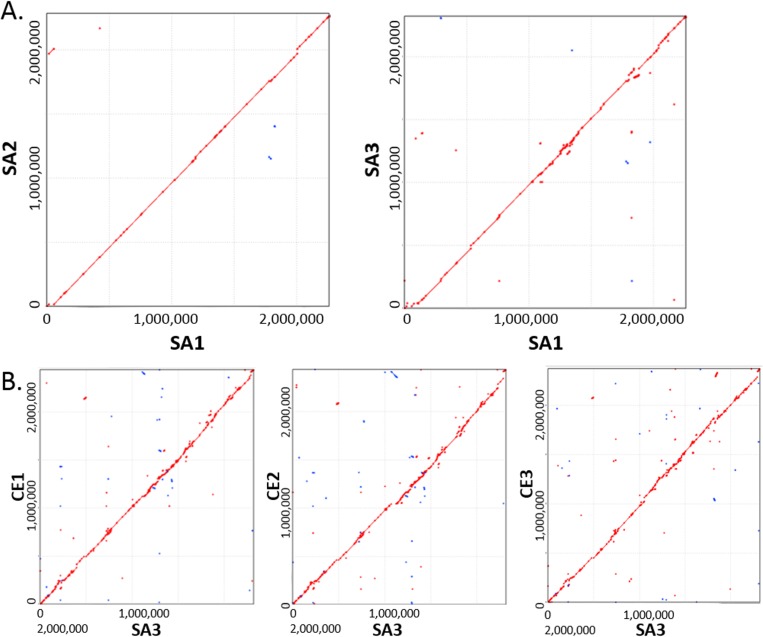
Synteny Plots of EC Chromosomes. Pathogenic EC genotypes (determined by PFGE) cluster tightly together compared to commensal EC. Genomes of pathogenic strains SA1, SA2, and SA3 are highly syntenic (A. SA1:SA2; B. SA1:SA3) which confirms clonality of these strains at high resolution. However, when compared to SA3, commensal strains CE1, CE2, and CE3 are much more diverse in genome content and arrangement (C. SA3:CE1; D. SA3:CE2; E. SA3:CE3).

Using MUMmer’s “nucmer” function, pair-wise comparisons of assembled SA1 and SA2 genomes revealed a high degree of synteny (Fig 1A). SA1 and SA2 sequences are nearly identical except for 2 small inversion events (in blue) near 1.8Mb and a translocation near the 2Mb region (Fig 1A). SA1 and SA2 have PFGE profiles that cluster closely together and genomic sequencing confirms that they are clonal at high resolution. SA3 has a PFGE profile within the pathogenic EC clade although it clusters less closely to SA1 and SA2.[9] Comparison of SA1 and SA3 revealed that, overall, the SA3 genome shares a high degree of synteny with SA1, but there are more differences than when SA1 is compared to SA2 (Fig 1B). Since the SA3 genome is larger than SA1 and SA2, it was selected to be the reference strain for comparisons with the non-pathogenic commensal isolates. Pairwise genomic alignments between SA3 and CE1, CE2, and CE3 are presented in Fig 1C–1E. Overall, these genomes were considerably more variable in chromosomal arrangement than pathogenic EC strains.

#### Molecular Phylogenetic Analysis

**Fig 2:**
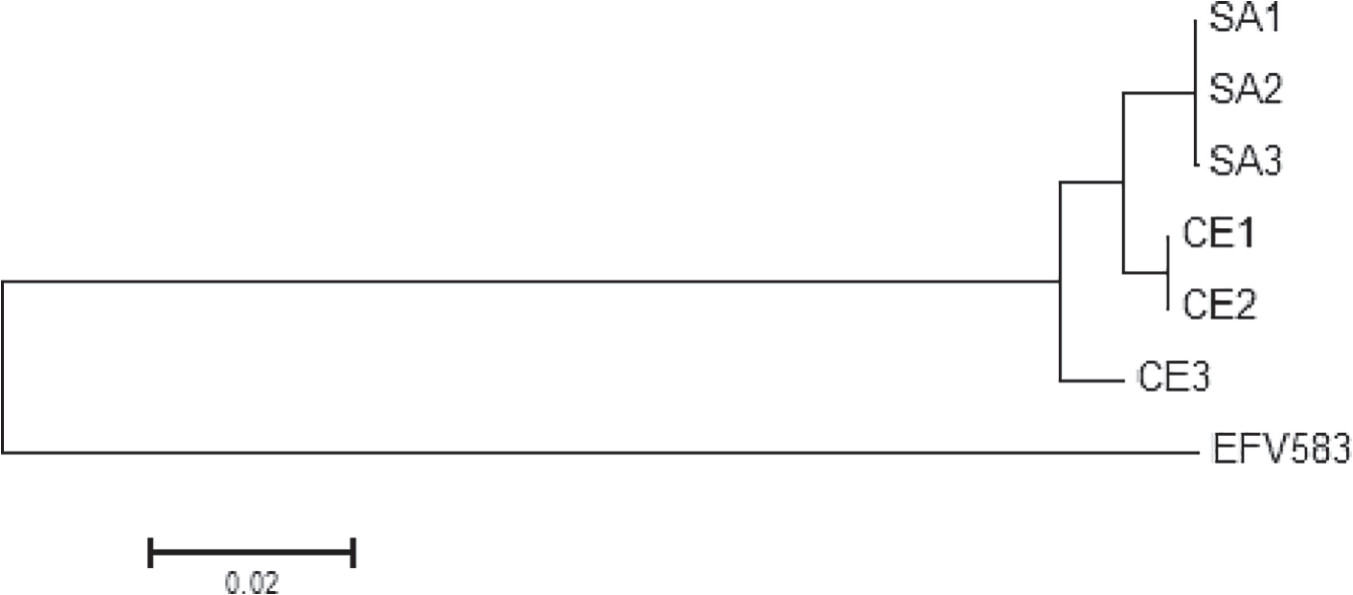
Molecular Phylogenetic Analysis of EC Genomes. The evolutionary history was inferred by using the Maximum Likelihood method based on the Tamura-Nei model. The tree with the highest log likelihood (-12083.9639) is shown. Initial tree for the heuristic search was obtained automatically by applying Neighbor-Join and BioNJ algorithms to a matrix of pairwise distances estimated using the Maximum Composite Likelihood (MCL) approach, and then selecting the topology with superior log likelihood value. The tree is drawn to scale, with branch lengths measured in the number of substitutions per site. The analysis involved 5 concatenated nucleotide sequences and used *E*. *faecalis* V583 as an outgroup. Evolutionary analyses were conducted in MEGA6.

Based on the structural differences in pathogenic and commensal EC, a molecular phylogenetic analysis was performed to assess for sequence divergence (Fig 2). For this analysis 5 core genes (*rrs*, *ddl*, *atpA*, *cpn60* and *sodA*) were selected based on their utility in speciation or classification of enterococci.[33–37] The phylogenetic analysis revealed divergence between the two groups of EC genomes.

#### CRISPR-*cas*

In addition, divergent CRISPR-*cas* systems were identified in pathogenic EC genomes. The CRISPR-*cas* organization in pathogenic strains follows the type I-C arrangement (*cas3*, *cas5*, *cas8c*, *cas7*, *cas4b*, *cas1*, *cas2*) which has been reported in streptococci but not in enterococci.[41–44] This locus is predicted to be active in SA1 and SA2 as they contain *cas1* and *cas2*.[45, 46] SA3, which has the largest of the pathogenic genomes, carries a similar type I-C arrangement although *cas2* was not identified suggesting that the locus may not be active in this strain. In contrast, non-pathogenic strains have the type II-A arrangement (*cas9*, *cas1*, *cas2*, *csn2*) that has been reported for enterococci. Absence of a type II-A CRISPR-*cas* system has been shown to be associated with increased drug resistance in medically important enterococci.[47, 48] The increased range of drug resistance previously observed in pathogenic EC may be due to this lack of a II-A CRISPR-*cas* system.[9] In addition, the divergent CRISPR-*cas* systems observed in pathogenic and commensal EC may provide insight into the evolution of pathogenic EC.

### Putative Virulence Determinants Conserved in Pathogenic EC

**Fig 3:**
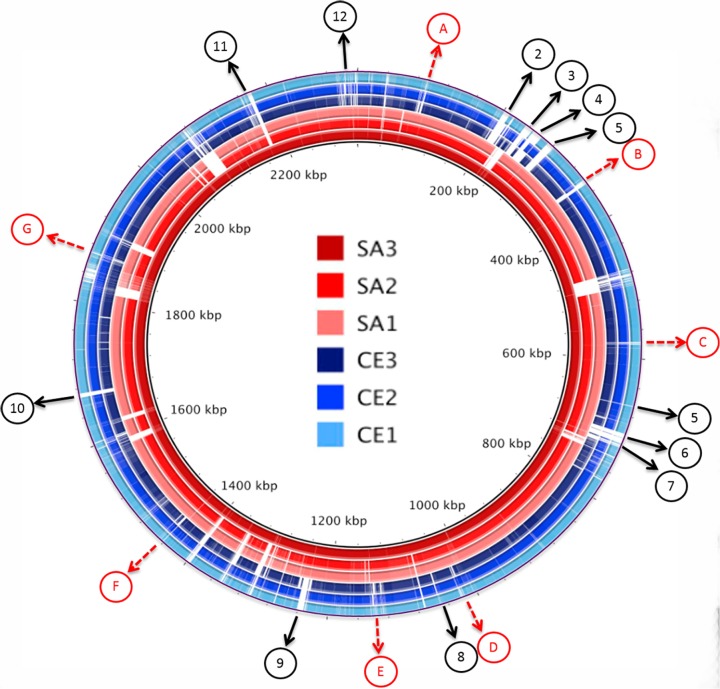
Genome Assemblies of EC Isolates Mapped Against Reference Sequence SA3. Scaffolds of all EC strains were mapped against strain SA3 (including SA3 as a control) using BRIG ring generator and BLASTn. Pathogenic EC strains SA1, SA2, and SA3 are presented in shades of red, commensal strains CE1, CE2, and CE3 are presented in shades of blue. Genetic sequences of less than 50% homology are plotted in white on each ring. 19 regions (labeled A-G and 1–12) are conserved in pathogenic EC, but not conserved in commensal EC (white areas). Regions A-G do not contain predicted coding sequence and represent inversions, rearrangements and insertions. ACT plots for these regions are provided in supplemental material (S2 Fig). The location on the SA3 chromosome map and the leading BLASTn result for each CDS are provided in supplemental material (S1 Table).

To identify chromosomal regions unique to pathogenic EC which might impact virulence, the BRIG tool was used with SA3 selected as the reference genome. The image produced by BRIG illustrates the circular chromosomes as concentric rings with the pathogenic strains SA1, SA2, and SA3 in red and commensal strains CE1, CE2, and CE3 in blue (Fig 3). White areas indicate locations where alignments between the individual sequences and the reference strain SA3 are < 50% identical. Nineteen of these regions with < 50% identity were selected for further investigation (Fig 3). Examination of these regions using ACT plots revealed that 7 do not contain coding sequences and genetic differences are due to a combination of inversions, deletions, and insertions (Fig 3A–3G and S2 Fig). The remaining 12 regions (Fig 3. 1–12) contain coding sequences that are conserved in SA1, SA2, and SA3 but share <50% identity in CE1, CE2, and CE3. The leading BLASTn result for each CDS and its location on the SA3 chromosome are provided in S1 Table.

**Fig 4:**
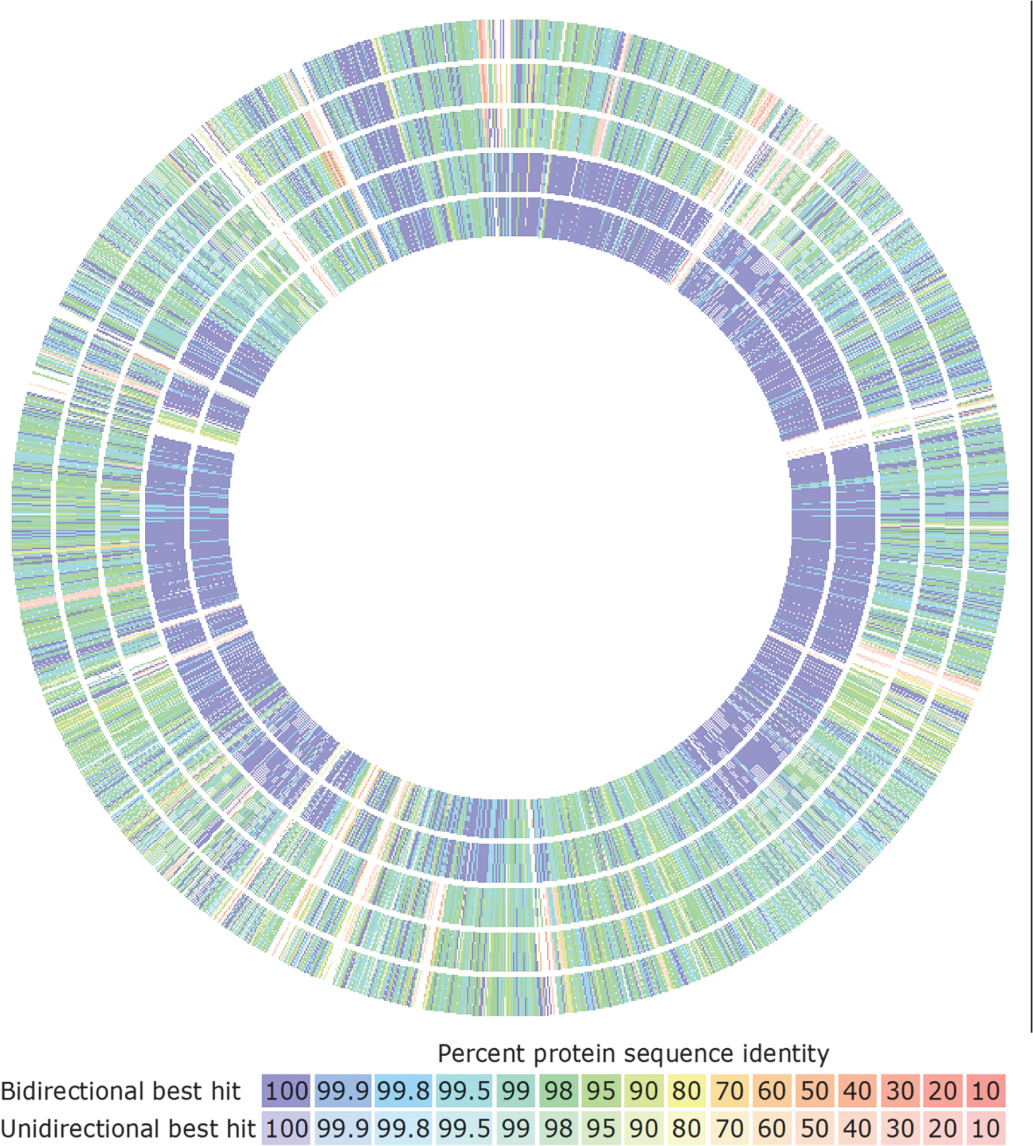
Comparison of Predicted EC Proteins. Protein sequences were compared using the RAST SEED viewer sequenced-based comparison tool with SA3 used as the reference sequence. The inner two rings are SA1 and SA2, while the outer three rings are CE1, CE2, and CE3 respectively. Percent protein sequence identity is expressed by color coding provided in the legend. A threshold of 98% sequence identity was used to identify sequences highly conserved in pathogenic strains compared to commensal strains. Using this threshold, a total of 414 predicted proteins were found to be highly conserved in pathogenic EC strains. The predicted function of these proteins and location on the SA3 chromosome are provided in S2 Table.

In a second approach, assembled genomes were uploaded to RAST for annotation and predicted proteins were compared using the SEED viewer (Fig 4).[31, 32] Filtering predicted proteins at a threshold of 98% sequence identity revealed 414 highly conserved predicted proteins in pathogenic EC that are not highly conserved in commensal EC (S2 Table).

**Fig 5:**
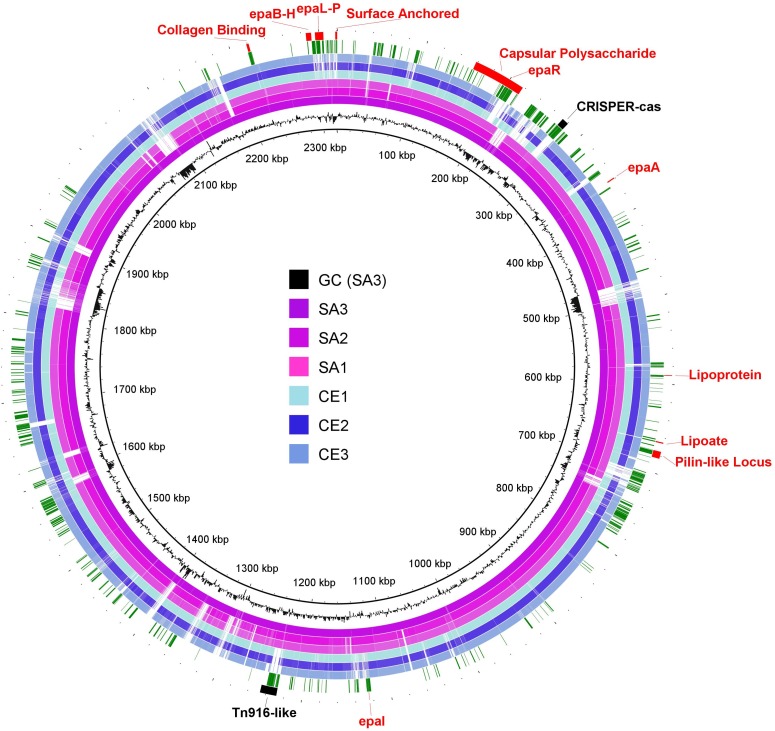
Predicted Proteins Highly Conserved in Pathogenic EC Mapped to Genome Assemblies of EC. Pathogenic (magenta) and commensal (blue) EC scaffolds were mapped to SA3 (including SA3 as a control) using BRIG ring generator and BLASTn. GC content of SA3 is plotted in black on the innermost ring. Increased GC content is represented by peaks toward the center of the circle and decreased GC content is represented by peaks away from the center. Genetic sequences of less than 50% homology are plotted in white on each ring. Outside the chromosomal rings and identified by green bars, are the genomic locations of the 414 predicted proteins with >98% identity in pathogenic strains and with <98% identity in commensal strains. Potential mediators of virulence are identified in red and include the capsular polysaccharide locus, *epa*-like genes and putative surface expressed proteins that may bind host substrates. Identified in black are the CRISPER-*cas* and Tn916-like regions.

These 414 conserved predicted proteins, including the CRISPR-cas and Tn916, were mapped to the SA3 genome (Fig 5), categorized by function to identify those that might impact virulence, and compared to closely related organisms including *E*. *faecalis* V583. Putative virulence factors identified included: a capsular polysaccharide locus, a gene cluster with homology to the enterococcus polysaccharide antigen (*epa*) locus, lipoproteins, and surface-anchored adhesins (Fig 5).

#### Capsular polysaccharide

In pathogenic strains, a polysaccharide capsule cluster was identified which is similar to the recently described capsule locus of *E*. *faecium*.[44] It is postulated that the capsular polysaccharide encoded by this locus may be responsible for the resistance to neutrophil-mediated phagocytosis observed in some strains of *E*. *faecium*.[49–51] Similar to *E*. *faecium*, all EC strains have 4 core genes, ECS3_0154, 0155, 0157, and 0158, which have homology to *Streptococcus pneumoniae* capsule genes *cps*ABCD and are oriented *cps*ACDB in EC (also designated *wzg*, *wzd*, *wze*, and *wzh* due to overlap in nomenclature in the enterococci).[36] ECS3_0155 encodes a putative manganese-dependent protein-tyrosine phosphatase which is homologous to CpsC of *S*. *pneumoniae* and shares 100% identity among pathogenic strains but 97.5% identity in non-pathogenic strains. A downstream extension of this locus contains an approximately 12kb region which encodes an array of polysaccharide biosynthesis genes which are highly conserved among pathogenic strains but have variable (97–20%) identity (or are absent entirely) from commensal strains (see S2 Table). Similarly within this region are phosphotransferases and other enzymes involved in carbohydrate metabolism which are highly conserved in pathogenic strains but have poor homology or are absent in non-pathogenic strains.

#### *epa*-like locus

**Fig 6:**
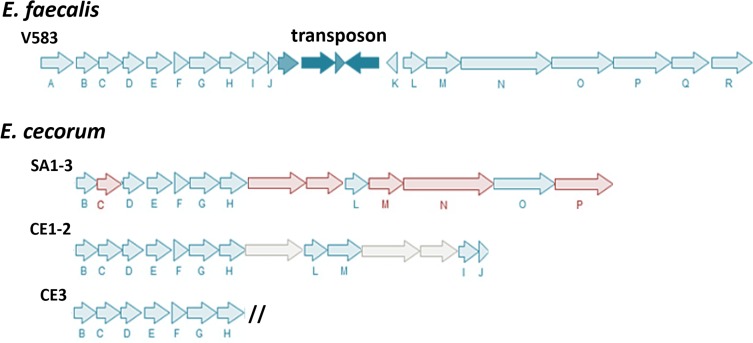
*epa*-like Locus of EC. *epa* orthologs identified in EC strains are presented as arrows labeled with letters that correspond to *epa*-gene orthologs. Orthologs found to be highly conserved in pathogenic EC are shaded in red. Pathogenic strains have a conserved chromosomal arrangement that is more similar to the *epa*-locus of *E*. *faecalis* V583 than commensal strains. The core *epa*-like locus which contains *epa*B-H is conserved among all strains; however, commensal EC strains have a variable arrangement downstream from this core region. Orthologs conserved in pathogenic strains: *epa*C, *epa*M, *epa*N, and *epa*P (red) are located within and downstream from the core *epa*-like locus. In all strains, orthologs to *epa*A (not shown) are found at distant genomic locations (see Fig 5). *epa*R and *epa*I (not shown) are conserved in pathogenic strains but are found in distant chromosomal locations (see Fig 5).

A gene locus similar to the enterococcal polysaccharide antigen (*epa*) locus of *E*. *faecalis* was identified in the sequenced strains and components of this locus were found to be highly conserved in pathogenic EC (Fig 6). Enterococcal polysaccharide antigen (Epa) is an immunoreactive, rhamnose-containing polysaccharide cell wall component which is an important mediator of enterococcal pathogenesis.[52] Epa is thought to be specifically involved in biofilm formation, tissue invasion, and resistance to killing by phagocytes.[53–57] In *E*. *faecalis* models of virulence, disruption of the *epa* locus is associated with attenuation and deficiencies in translocation across a polarized monolayer of colonic epithelial cells.[54, 55, 57]

In *E*. *faecalis* the *epa* locus is an 18 gene (*epa*A-R) cluster which is modified in *E*. *faecium* to a 15 gene cluster (*epa*A-H,P,Q,L-R).[53, 56, 58, 59] In pathogenic and commensal strains, the core *epa*B-H genes are arranged in a cluster and share 93 to 36% identity to orthologs in *E*. *faecalis* V583 (S3 Table). Orthologs to *epa*A and *epa*R were identified at separate distant genomic locations in all 6 strains. In general, the arrangement of the *epa*-locus of pathogenic strains more closely mirrors the arrangement of *epa* genes in *E*. *faecalis* V583 (Fig 6). In pathogenic strains, orthologs to *epa*L-P with 73 to 31% identity to *E*. *faecalis* V583 are separated from the *epa*B-H core by a predicted lysozyme M1 (1,4-beta-N-acetylmuramidase) (EC 3.2.1.17) and a glycosyltransferase. These 2 inserted genes are highly conserved (99.3 and 100% identity) among pathogenic strains. Homologous lysozyme and glycolsyltransferase genes (62.0–42.6% and 24.7% identity respectively) were identified in distant genomic locations in commensal EC. Several individual *epa*-locus genes were identified by our analysis as conserved in pathogenic EC including: *epa*C, *epa*M, *epa*N, *epa*P and *epa*R-like genes (100–98.91% identity). Pathogenic strains lacked an *epa*J ortholog and an *epa*I-like gene was identified at a distant genomic location in these strains.

In contrast to pathogenic strains, the region downstream from the *epa*B-H core varied among commensal strains. CE1 and CE2 share a similar arrangement with orthologs to *epa*I, *epa*J, *epa*L, and *epa*M identified within this region while CE3 has an approximately 45kb insertion in this region. Additional *epa*-locus orthologs arranged *epa*M, *epa*I, *epa*J, *epa*L, *epa*O and *epa*R follow this insertion in CE3. These orthologs are not contained in an operon and are interspersed with genes with varied predicted function. In all non-pathogenic strains, an ortholog to *epa*N was identified at a distant site within the genome and orthologs to *epa*P and *epa*O were not identified in these strains.

#### Host binding proteins

Surface expressed proteins which bind host tissues are major components of enterococcal virulence.[60–65] Putative surface expressed proteins were identified in the sequenced strains including predicted proteins with homology to collagen-binding proteins, fibronectin-binding proteins, lipoproteins and proteins containing a cell wall targeting LPXTG domain with unknown function.

Proteins with predicted collagen binding type B domains were identified in pathogenic and commensal strains and several of these proteins were highly conserved in pathogenic strains. A gene cluster reminiscent of the endocarditis and biofilm-associated pili (Ebp) pilus-encoding locus of *E*. *faecalis* was identified in EC. The Ebp pilus-encoding region of *E*. *faecalis* consists of a 3 gene locus, *ebp*ABC (EF1091-1093) followed by the pilus-associated sortase (bps) EF1094.[66, 67]. In EC, this gene cluster is composed of a putative ArsR-family transcriptional regulator followed by 3 cell wall surface-anchor-family proteins with collagen binding protein type B repeat domains and an array of 3 type A sortases. The transcriptional regulator (ECS3_0674) and the first 2 putative surface-anchor-family proteins, ECS3-0675 and ECS3-0676 have identical protein sequence in pathogenic EC but have ~90 to 97% identity in commensal EC. Of these, ECS3-0676 is an *ebp*C ortholog and contains a fimbrial isopeptide formation D2 domain as well as collagen binding protein type B repeat domains. Homologous surface-anchored proteins are well characterized virulence factors of Gram positive pathogens, which are frequently involved in adhesion to host structures.[68, 69] [40]

In a separate 4kb region there are 6 hypothetical proteins which are highly conserved in pathogenic EC (99 to 100% protein sequence identity) but have a more diverse protein sequence in commensal EC (absent to 97% identity). Of these, 2 (ECS3_2204 and ECS3_2205) contain predicted collagen binding domains with homology to a collagen binding protein of *Clostridium perfringens*.

A highly conserved predicted lipoprotein (ECS3_0596) was identified which had identical protein sequence in pathogenic EC and shared ~96% protein identity with commensal EC. In addition, pathogenic EC have a conserved gene cluster (ECS3_0662–0664) that contains a predicted lipoate synthase andligase, which is absent in commensal EC, and a lipoate transferase which shares ~97% protein identity with an ortholog in commensal EC strains.

In addition, a putative surface anchored protein ECS3_2316 is conserved in pathogenic EC, absent in commensal EC, and is homologous to EF0109, a surface expressed protein of unknown function that is found in the pathogenicity island of *E*. *faecalis*.[70, 71]

### Virulence Genes Common to Pathogenic and Commensal EC

A putative fibronectin-binding protein (FBP) was identified in both pathogenic and non-pathogenic strains and is homologous to a FBP that occurs in several species of enterococci.[60]

Two predicted proteins demonstrated significant homology to hemolysin III (ECS3_0622) and hemolysin A (ECS3_1931). While hemolysins function in the virulence of several Gram positive organisms including enterococci; these putative hemolysins are conserved among both pathogenic and commensal strains.[65]

The superoxide dismutase (*sod*A) gene (ECS3_0614) is conserved among all strains. This was expected as nucleotide sequence variation in the encoding gene has been exploited for enterococcal species identification using multiplex PCR.[72] SodA detoxifies the oxygen free radicles produced by the oxidative burst and has been shown to blunt macrophage killing of enterococci. [73]

### Conserved Genome Features of Commensal EC

**Fig 7:**
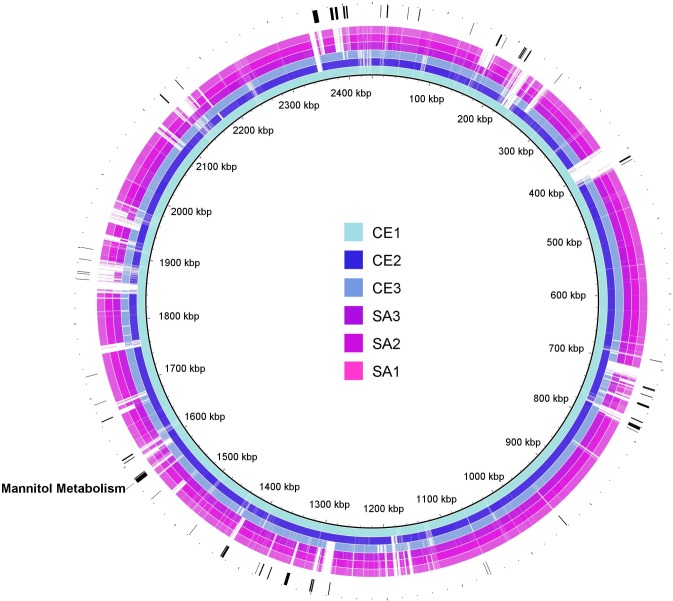
Predicted Proteins Conserved in Commensal EC Mapped to Genome Assemblies of EC. Pathogenic (magenta) and commensal (blue) EC scaffolds were mapped to CE1 (including CE1 as a control) using BRIG ring generator and BLASTn. Genetic sequences of less than 50% homology are plotted in white on each ring. Outside the chromosomal rings and identified by green bars, are the genomic locations of the 95 predicted proteins with >90% identity in commensal strains and with <90% identity in pathogenic strains. Genomic predicted proteins involved in mannitol metabolism are labeled in black.

As pathogenic EC genomes were significantly smaller than commensal EC genomes; a second analysis was performed to identify predictive proteins that were conserved in commensal EC but not in pathogenic EC. To identify chromosomal regions unique to commensal EC, the BRIG tool was again used but with CE1 (the largest commensal genome) as the reference genome (Fig 7). Predicted protein sequence identity was compared among EC strains using RAST and the CE1 genome as the reference. However, given the relative diversity of commensal EC, a less stringent threshold of 90% was set to identify conserved predicted proteins in commensal EC. Using this threshold, 95 predicted proteins were identified with >90% sequence identity in CE1, CE2, and CE3 and <90% identity in SA1, SA2, and SA3 (S4 Table). These 95 predicted proteins were mapped to the CE1 genome in Fig 7.

**Fig 8:**
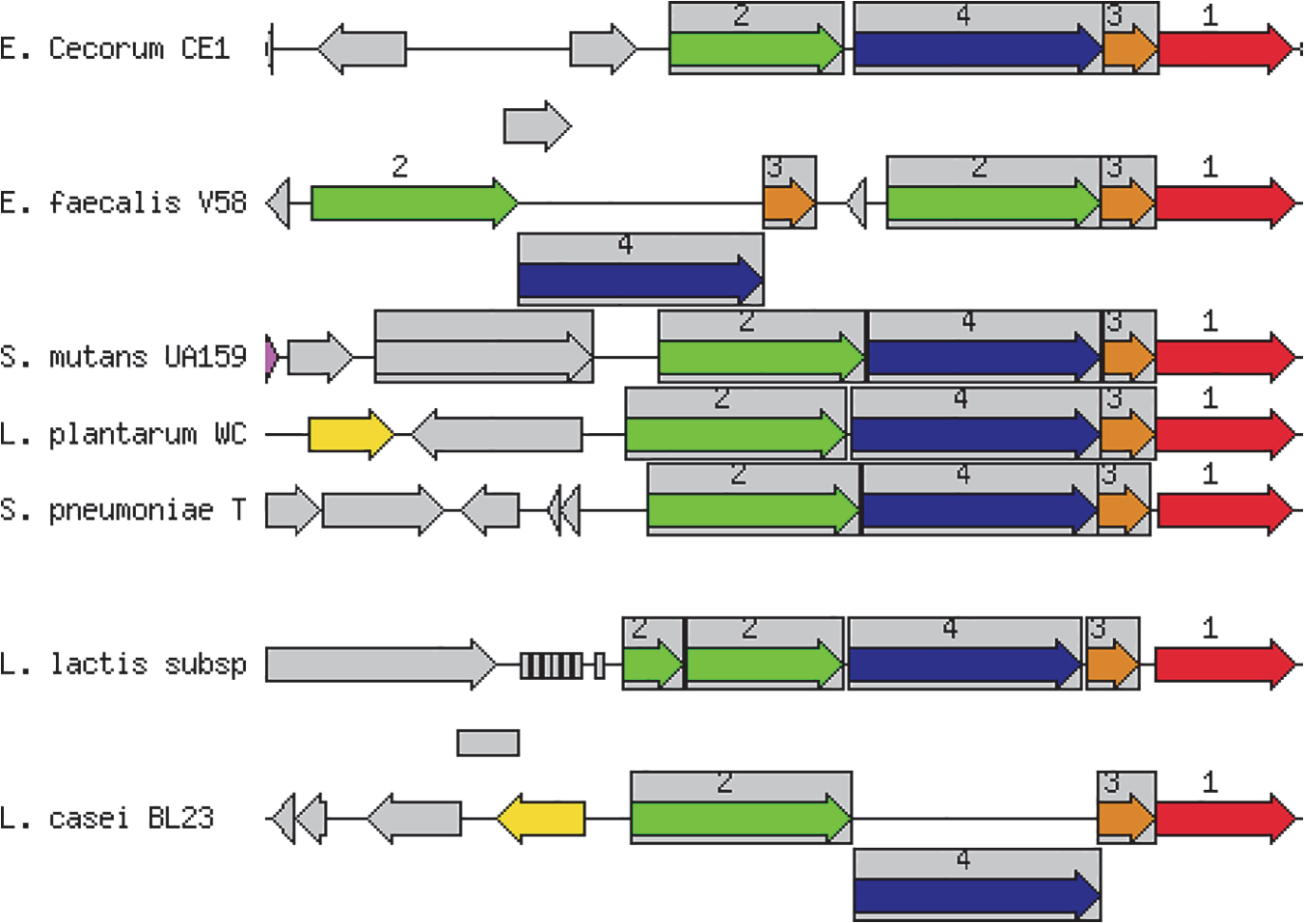
Mannitol Metabolism Locus of Commensal EC. The chromosomal region surrounding the conserved mannitol utilization locus of commensal EC is compared with 2 streptococci, 3 lactococci and E. faecalis V583. Othologous genes are grouped with the same number and color. Genes whose relative position is conserved in at least four other species are functionally coupled and share gray background boxes. From left to right the mannitol utilization genes are oriented as follows: mannitol-specific PTS system IIB/IIC which is labeled in green and numbered 2; the mannitol operon activator, BglG family which is labeled in orange and numbered 3; the IIA component of the mannitol-specific PTS system which is also labeled in green and numbered 2; and the mannitol-1-phosphate 5-dehydrogenase which is labeled in red and numbered 1. Interestingly, the arrangement of this mannitol utilization locus in commensal EC is more similar to the arrangement in streptococci and lactococci than E. faecalis V583.

Many of the 95 identified predicted proteins have unknown function, but of those with predicted function, many are involved in carbohydrate metabolism. Most notable of these are predicted mannitol-specific phosphotransferase system (PTS) components, mannitol operon activator (BglG family) and a mannitol-1-phosphate 5-dehydrogenase (ECC1_0810–0812). The PTS components and operon activator share low (~25%) sequence identity with predicted proteins in pathogenic EC and the predicted mannitol dehydrogenase enzyme is absent in pathogenic EC. The arrangement of orthologous genes in commensal EC is similar across several species of streptococci and lactococci but is different from the arrangement in *E*. *faecalis* V583 (Fig 8). A defect in mannitol metabolism has been established as a feature of pathogenic EC.[9, 14] It is likely that this region, which is conserved in commensal strains but not pathogenic strains, explains this observation.

## Conclusions

Recently emerged pathogenic strains of EC represent a significant threat to broiler chicken production worldwide. In a prior study, pathogenic strains from the southeast US were shown to be genetically clonal, defective in mannitol metabolism, and have an increased range of antimicrobial resistance. In this work, comparison of pathogenic and commensal genomes revealed that pathogenic EC from the southeast US are clonal at high resolution and have divergent genomic features. Pathogenic EC have significantly decreased overall genome size and have a higher GC content. In a molecular phylogenetic analysis using 5 genes commonly used to speciate enterococci, sequenced pathogenic EC strains formed a separate clade further suggesting divergent evolution of these strains. In addition, a type I-C CRISPR-*cas* system was identified in pathogenic EC which was predicted to be functional in 2 of the 3 pathogenic strains. The type I-C arrangement is not typical for enterococci but has been identified in streptococci. As the type IIA arrangement typical of enterococci was found in commensal strains, variation in the CRISPR-*cas* systems may prove useful to identify pathogenic strains and to investigate their divergent evolution. Finally a molecular basis for mannitol metabolism was observed in commensal EC. This locus was absent in pathogenic EC which likely explains early observations regarding the inability of pathogenic strains to use mannitol.[9, 14]

Several potential mediators of virulence were found to be highly conserved in pathogenic EC. A polysaccharide capsule locus was identified in pathogenic EC which is similar to a novel capsular locus recently reported in other enterococci.[44] Likewise, an *epa*-like locus was found to be highly conserved in arrangement and protein sequence identity in pathogenic EC. Epa is immunogenic in other medically important enterococci and conservation of the *epa* locus in pathogenic strains suggests that the encoded carbohydrate antigen may be important for virulence or may be under selective pressure during infection. The capsular polysaccharide and *epa* locus identified by this analysis are thought to mediate evasion of phagocytic killing in other medically important enterococci.[49–51, 53–57] As such, these virulence determinants may play a role in the apparent inability of affected birds to control infection by pathogenic EC. Finally, several putative surface expressed proteins with homology to enterococcal and clostridial collagen binding proteins were found to be highly conserved in pathogenic EC. Binding to collagen in bone or cartilage of the vertebral column may partially explain the characteristic lesion localization of pathogenic EC.

In conclusion, comparison of EC genomes revealed fundamental differences in pathogenic EC genomes and identified potential virulence determinants. While these findings are consistent in the 3 sequenced pathogenic strains that represent isolates from the southeast US; it remains unclear if there is regional diversity in pathogenic EC. Outbreaks of pathogenic EC occur throughout the US broiler system and it is yet to be determined if the pathogenic EC possess regional genotype diversity. Comparative genomic analysis of additional, genetically diverse, pathogenic EC strains from across the US broiler system would be useful to support the conclusion that specific virulence determinants identified by this study contribute to pathogenicity of EC. In general, the virulence determinants conserved in pathogenic EC were found to be similar to those utilized by other medically important enterococci. Therefore the recent emergence of pathogenic EC strains provides a unique opportunity to identify virulence mechanisms of enterococci and reduce their impact on human and animal health.

## Supporting Information

S1 Fig GC Content and Skew for SA3. Using BRIG, the GC content and skew of the SA3 genome are presented. Increased GC content and positive skew are represented by peaks oriented toward the center of the circle. Decreased GC content and negative skew are represented by peaks away from the center. GC content and skew appear randomly distributed throughout the majority of the genome with the exception of a 400kb region from 900Kb to 1300Kb of decreased GC content and negative GC skew.
(TIF)

S2 Fig ACT Plots of Genomic Regions A-G. ACT plots of genomic regions A-G reveal insertions, deletions and rearrangements in regions without CDS.
(TIF)

S1 Table Genomic Location and BLASTn Result for Regions 1–12. The genomic location on the SA3 genome and the leading BLASTn result for each CDS are provided.
(XLSX)

S2 Table Genomic Location, Annotation of Percent Sequence Identity of 414 Predicted Proteins Conserved in Pathogenic EC. The genomic location on the SA3 genome, predicted function and percent identity across all sequenced EC strains for each of the 414 conserved CDS are provided. Gene locations of potential virulence factors conserved in pathogenic EC and the CRISPR-*cas* region are highlighted in red or black (respectively) and correspond to similarly colored annotations on the BRIG chromosomal alignment in Fig 5. Fields with percent identity are color coded as in Fig 4.
(XLSX)

S3 Table Orthologs in the *epa*-Locus. The genomic location on the SA3 genome and annotation for *epa*-locus orthologs are provided. Individual *epa*-locus percent identity to *E*. *faecalis* V583 and SA3 orthologs is provided and color coded as in Fig 3.
(XLSX)

S4 Table Genomic Location, Annotation, Percent Sequence Identity of 95 Predicted Proteins Conserved in Commensal EC. The genomic location on the CE1 genome, predicted function and percent identity across all sequenced EC strains for each of the 95 identified predicted proteins are provided. The predicted mannitol-specific PTS system and associated mannitol dehydrogenase are highlighted green. Fields with percent identity are color coded as in Fig 4.
(XLSX)
