# Supplementary figures and images for: Comparative Genomic Analysis Identifies Divergent Genomic Features of Pathogenic Enterococcus cecorum Including a Type IC CRISPR-Cas System, a Capsule Locus, an epa-Like Locus, and Putative Host Tissue Binding Proteins
Source: PLoS One. 2015 Apr 10;10(4):e0121294. doi: 10.1371/journal.pone.0121294 (PMC4393107; doi:10.1371/journal.pone.0121294)

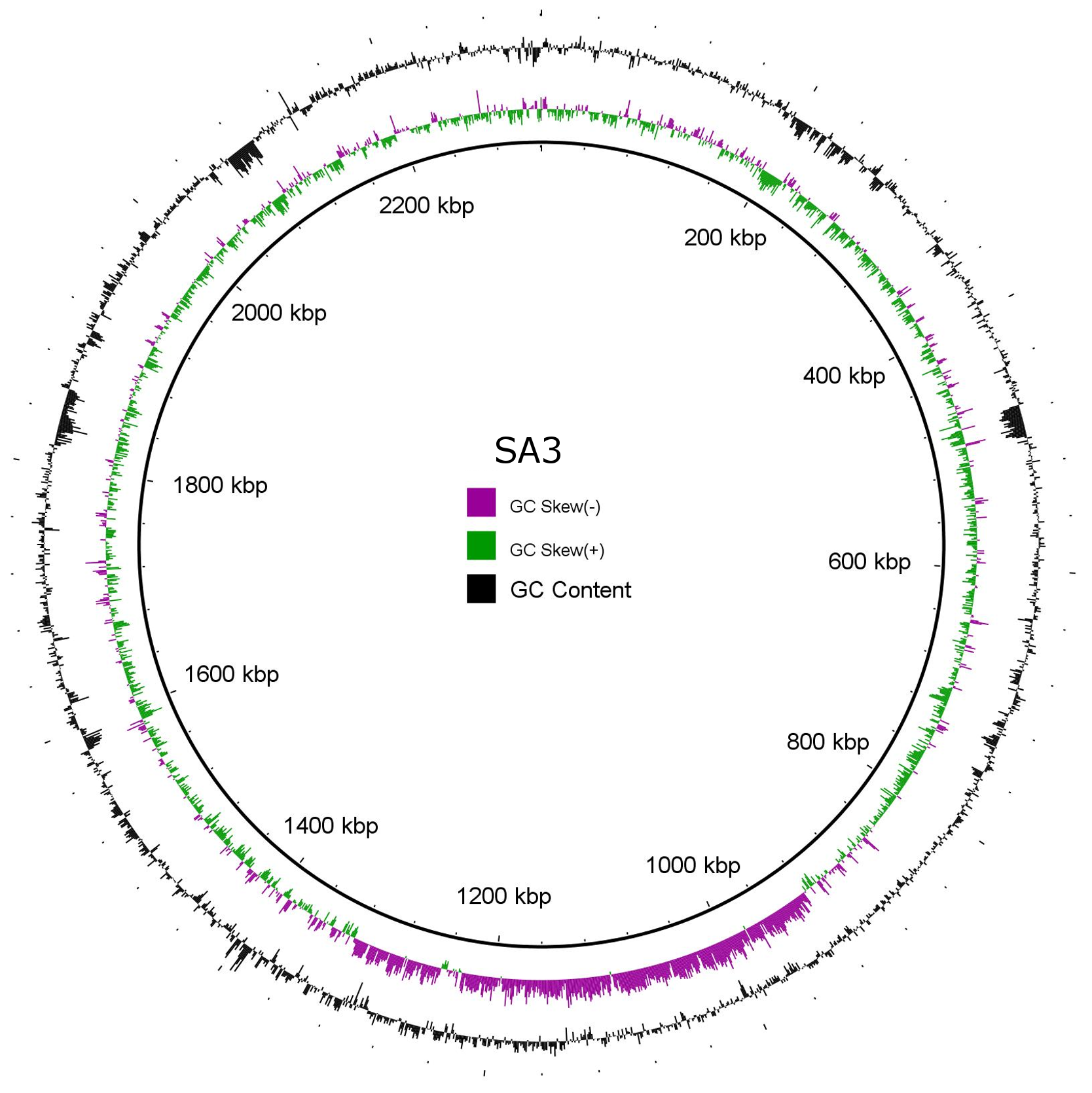

Supplement: S1 Fig — Using BRIG, the GC content and skew of the SA3 genome are presented. Increased GC content and positive skew are represented by peaks oriented toward the center of the circle. Decreased GC content and negative skew are represented by peaks away from the center. GC content and skew appear randomly distributed throughout the majority of the genome with the exception of a 400kb region from 900Kb to 1300Kb of decreased GC content and negative GC skew. (TIF) [file pone.0121294.s001.tif]

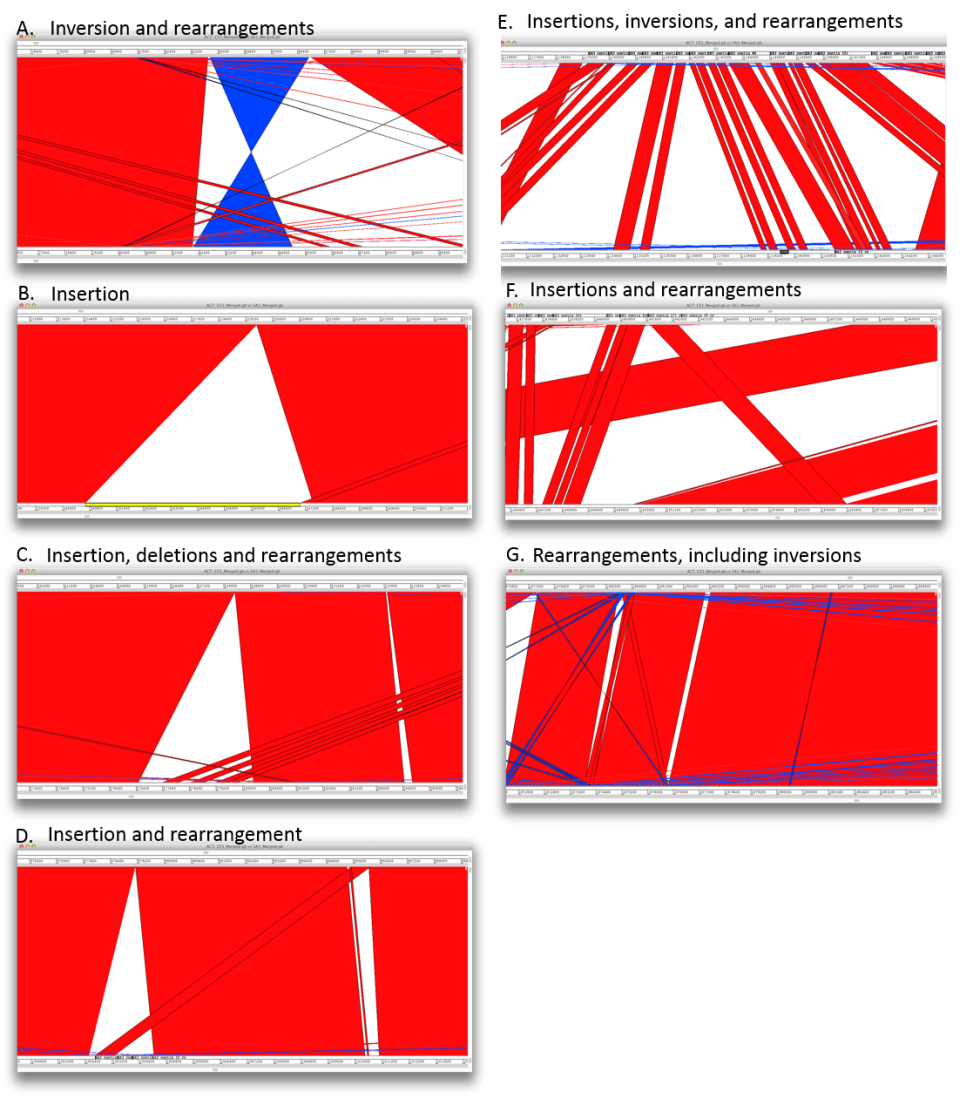

Supplement: S2 Fig — ACT plots of genomic regions A-G reveal insertions, deletions and rearrangements in regions without CDS. (TIF) [file pone.0121294.s002.tif]
